# Tooth Root Abscess and Mandibular Infections in Old World Camelids: 3 Cases

**DOI:** 10.1155/2024/4589572

**Published:** 2024-04-02

**Authors:** Alyssa Sparnon, Joe Smith, Pierre-Yves Mulon, Silke Hecht, David Anderson, Sarel Van Amstel

**Affiliations:** ^1^Large Animal Clinical Sciences, University of Tennessee College of Veterinary Medicine, Knoxville, USA; ^2^Small Animal Clinical Sciences, University of Tennessee College of Veterinary Medicine, Knoxville, USA

## Abstract

There are limited reports of mandibular infections and tooth root abscesses in camels (Old World Camelids). This is in contrast to multiple reports and case series detailing diagnosis, management, and therapy of similar infections in New World Camelids such as llamas and alpacas. The purpose of this case series is to present three cases of camels in North America with these infections and to detail the diagnostics, therapeutic interventions, management, and follow-up of these cases. Radiography was utilized in all three cases, as was sedation and/or anesthesia. Similar to llamas, florfenicol was used for antimicrobial therapy and flunixin meglumine was utilized as a nonsteroidal anti-inflammatory. Some degree of lavage was required for the management of each case. Clinicians should be aware of the potential need for sedation, diagnostic imaging, culture, and extended therapies for the treatment of mandibular and tooth root infections in camels as comparatively described for llamas and alpacas.

## 1. Introduction

Currently, there is a paucity of case reports of mandibular infections in Old World Camelids. In contrast, South American camelids have commonly presented to veterinarians for dental disease that is often caused by tooth root abscessation and mandibular infections [1]. Although native to regions such as the Middle East, Africa, and Asia, the numbers of dromedary (*Camelus dromedarius*) and Bactrian (*Camelus bactrianus*) camels have started to increase in recent years in both Australia and North America [2, 3]. Veterinarians are increasingly asked to provide health care for camels, including treatment of dental disease. Unfortunately, there is a dearth in the literature describing mandibular infections in camels.

In South American camelids, mandibular infections originating from tooth root infections most often present with hard swellings surrounding the affected teeth. Published studies of cases occurring in llamas and alpacas have reported that mandibular teeth are more likely to develop a tooth root abscess than maxillary teeth. Although a definitive predisposing factor has not been found, it is thought that a combination of factors including stemmy stored forages, diets including concentrates, altered grazing patterns in fenced pasture management, and aging are associated with irritation in the oral mucosa and disruption of the periodontal tissues most likely resulting in tooth root abscess [1]. One study regarding dental disease in camels in Nigeria found that dental disease was the most common factor associated with decreased life spans in these animals, causing them to be unthrifty and referred to as “poor doers” [4]. With regard to llamas and alpacas, it has been discussed that many of these animals present for a mandibular swelling but do not seem to be in pain while masticating.

The purpose of this study is to perform a retrospective study on camels presented to the Charles and Julie Wharton Large Animal Hospital at the University of Tennessee College of Veterinary Medicine during the time period inclusive of 2000 and 2023 for mandibular infection. The limited number of case reports regarding tooth root abscesses in Old World Camelids creates a need to describe the presentation, diagnosis, and management of these cases for the benefit of clinicians presented with cases in the future.

## 2. Case Presentation

A retrospective search was done of the UTVMC record database for all camel cases from 1980 to present. Search functions included a designation of camel as species, description of a tooth root abscess, and/or the coded diagnosis. Records were checked for the diagnosis of tooth root abscess and then further evaluated. Cases that lacked a clear diagnosis were excluded.

### 2.1. Case 1

A six-month-old intact female dromedary camel presented for a 1.5-week duration of local mandibular swelling over left mandible. Initially, the swelling was only noticed because there was purulent material draining from the left mandible. The attending veterinarian provided instructions for treatment with penicillin (procaine penicillin G, dose and frequency unknown) which was administered by the owners over the course of four days. Little to no response was appreciated by the owners, and this progressed to increased swelling of the soft tissues. The camel was referred for further diagnostics to the Charles and Julie Wharton Large Animal Hospital.

Upon initial examination, the camel weighed 201 kilograms and had a 5-centimeter diameter mass on the ventral portion of the left mandible. Purulent material could be expressed when pressure was applied. All other parameters were within normal limits.

**Figure 1:**
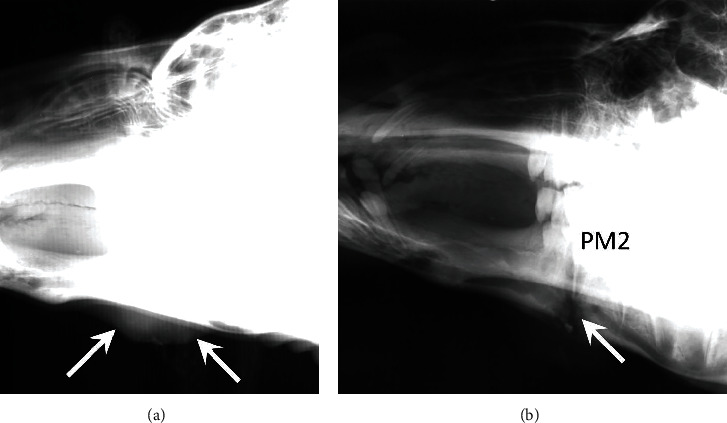
(a) Lateral and (b) right ventral-left dorsal oblique view of the left mandible in patient 1. Radiograph (a) was obtained with a technique optimized for soft tissue evaluation, and radiograph (b) was obtained with a technique optimized for bone evaluation. The radiographs were obtained with a conventional film-screen radiography system, and the radiographs were digitized for publication. (a) There is a focal lobular area of soft tissue swelling with a superficial defect present ventral to the left mandibular body (arrows). (b) At the level of the soft tissue swelling, there is a linear radiolucent tract (arrow) extending through the ventral mandibular cortex in a dorsal and rostral direction, reaching the rostral tooth root of the second mandibular premolar tooth (PM2). This tract is irregularly marginated and bordered by regions of medullary sclerosis and lamellar periosteal new bone formation along the ventral mandibular cortex. A pinpoint mineral opaque fragment present in the most ventral aspect of the tract represents either a small bone fragment or debris.

The camel was sedated with a combination of ketamine (5 mg/kg, intravenously, once) and xylazine (0.5 mg/kg, intravenously, once) before radiographs of the head were performed. Lateral and right ventral-left dorsal oblique views were taken. A focal lobular area of soft tissue swelling with a superficial defect was present ventral to the left mandibular body (Figure 1(a)). At the level of the soft tissue swelling, there was a linear radiolucent tract extending through the cortex of the ventral aspect of the horizontal ramus of the mandible, extending in a dorsal and rostral direction, and ultimately reaching the rostral tooth root of the second mandibular premolar tooth (Figure 1(b)). This tract was irregularly marginated and bordered by regions of medullary sclerosis and lamellar periosteal new bone formation along the ventral mandibular cortex. A focal mineral opacity fragment was present in the most ventral aspect of the tract, either representing a small bone fragment or debris. While being sedated, the soft tissue area was incised and a sample for culture was obtained that later revealed moderate branching Gram-positive rods, rare Gram-negative rods, and Gram-positive cocci. Final microbiology results were *Corynebacterium* sp. and *Actinomyces* sp., as well as two unidentified anaerobes, a Gram-positive cocci and a Gram-negative rod. Increased soft tissue swelling was noted around the second mandibular premolar, and it was removed. The incision was then lavaged with betadine before being left open to allow for drainage, and florfenicol (40 mg/kg, subcutaneously, every 96 hr) was administered. Recovery from sedation was uneventful. Lavage was achieved by deep intralesional flushing through the opening, daily, until the incision was filled with granulation tissue.

The camel was discharged with instructions to continue florfenicol and return to the Charles and Julie Wharton Large Animal Hospital for re-evaluation three weeks later. Examination indicated that healing was sufficiently progressive and that she needed only to be rechecked if owners were concerned or if clinical signs persisted. The camel was continued on florfenicol for one additional week after the recheck. Clinical resolution of the mandible infection was uneventful, and the camel participated successfully to the farm breeding program during the following years before being euthanized 8 years later for an unrelated condition.

### 2.2. Case 2

A four-year-old intact female Bactrian camel presented for a mandibular swelling that had progressed over several months (exact duration unknown). Initial exam revealed that the camel weighed 350 kilograms but seemed underweight and in poor condition which included slumping of the dorsal humps. A swelling over the left mandible was noted, all other vitals were within normal limits.

The camel was sedated with ketamine (3 mg/kg, intravenously, once) and xylazine (0.2 mg/kg, intramuscularly, once) so that oblique lateral and dorsoventral head radiographs could be done. Radiographs were not available for review at the time of manuscript construction, as the images were not saved from the conversion from film to digital radiography. Interpretation of the radiographic images reported radiolucency surrounding the second lower left cheek tooth (presumable the 4th mandibular premolar tooth). While being sedated, the affected area was incised and flushed with betadine after samples for bacterial culture obtained. Culture later revealed the presence of Gram-positive cocci and *Trueperella pyogenes*. The camel was treated with florfenicol (20 mg/kg, subcutaneously, every 72 hours) postoperatively, as well as flunixin meglumine (1 mg/kg, subcutaneously, once). Recovery from sedation was uneventful.

The camel was discharged with instructions to continue florfenicol and directions to flush the incision with betadine daily as described for case 1. Three weeks later, the camel returned for a scheduled re-evaluation and the lesions were resolving satisfactorily. The camel was continued on florfenicol at home.

### 2.3. Case 3

A three-year-old intact male dromedary camel presented after having a history of right mandibular abscessation. The camel had a six-month history of an abscess being present and draining while at home. The camel was prescribed a trimethoprim-sulfamethoxazole (TMS) powder (dose unknown) from the primary veterinarian with instructions for the owners to add into his food daily.

**Figure 2:**
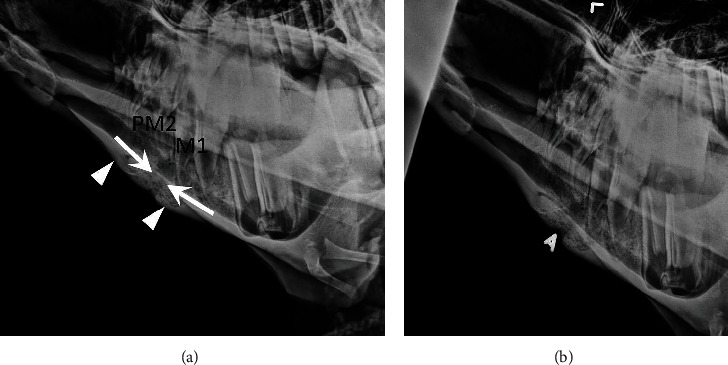
(a, b) Two separate left ventral-right dorsal oblique projections of the right mandible in patient 3. A metallic marker has been placed at the location of a clinically reported draining tract in (b). There is a radiolucent tract (arrows) extending from the ventral cortical margin of the mandible in a dorsal and caudal direction to reach the caudal tooth root of the second right mandibular premolar tooth (PM2) and the rostral tooth root of the right first mandibular molar tooth (M1). This tract is bordered by medullary sclerosis and associated with extensive relatively smooth periosteal new bone formation along the ventral body of the mandible (arrowheads). The clinically reported soft tissue swelling along the ventral mandibular body is unable to be evaluated radiographically due to technique. Note: the circular lucent region associated with the caudal mandible is consistent with a normal tooth bud of the second mandibular molar tooth. In camels, the tooth bud of the 2^nd^ mandibular molar begins to form at 1.5 years of age, and the tooth erupts at 3 years (approximate time of radiographs in this patient). Formation of this tooth is not complete until 4.5 years, and the tooth bud will not completely resorb until this age has been reached.

Upon initial examination, the camel was bright and alert. The location of the abscess was on the center portion of the right mandible, approximately 10 cm in diameter. At the time of admission, the body weight was estimated at 450 kilograms. Despite the ruptured abscess present on his right mandible, the rest of his physical exam was unremarkable. Before radiographs of the head were taken, the bull was sedated with xylazine (0.22 mg/kg, intramuscularly, once). Lateral, dorsoventral, and oblique radiographs of the head were obtained and showed a radiolucent tract extending from the ventral cortical margin of the mandible in a dorsal and caudal direction to reach the caudal tooth root of the right mandibular 2^nd^ premolar and the rostral tooth root of the right first mandibular molar tooth (Figure 2). This tract was bordered by medullary sclerosis and associated with extensive smooth periosteal new bone formation along the ventral body of the mandible. The clinically reported soft tissue swelling was unable to be evaluated radiographically due to technique.

Therapy was initiated with sodium iodide (60 mg/kg, intravenously, once weekly) diluted into 1 liter of balanced polyionic fluids. Two days later, the bull was induced into general anesthesia using xylazine (0.22 mg/kg, intravenously, once), ketamine (3 mg/kg, intravenous, as a bolus, followed by a 2.4 mg/kg/hr constant rate infusion-CRI), and diazepam (0.1 mg/kg, intravenous, once). The bull was maintained under general anesthesia using a ketamine CRI, morphine (0.15 mg/kg, intravenous over 30 minutes), and lidocaine (3 mg/kg intravenously as a loading dose over 15 minutes, followed by a 6 mg/kg/hour CRI) and supplemented with isoflurane gas vaporized into 100% oxygen. A curvilinear incision was made into the skin and subcutaneous tissue of the center of the right mandible. After opening the incision, two samples from the infected area were submitted for culture that later revealed bacteria growth including Gram-positive rods (noted on the microbiology report as “*Actinomyces*-like”). The periosteum was then retracted in order to remove a section of excess bone proliferation as to widen the opening to increase drainage and expose an area for flushing of betadine. The incision was closed except for an approximately 2.5-centimeter space that was left open to allow for drainage. Intraoperative radiographs were obtained and revealed no change in the previous appearance. Recovery from anesthesia was uneventful.

Treatment was initiated by administration of flunixin meglumine (1.0 mg/kg, intravenously, every 24 hours) postoperatively for five doses, as well as oxytetracycline (10 mg/kg, diluted into 1 liter of balanced polyionic fluids intravenously, every 24 hours). The camel was discharged with instructions to administer 6-10 grams of potassium iodide on top of feed for a week as well as florfenicol (20 mg/kg, subcutaneously, every 24 hours, for 4 doses).

## 3. Discussion

There are a multitude of mandibular infections documented in llamas and alpacas [1]; however, the information for camels is lacking. Mandibular infections can cause systemic health to decline and can result in hyporexia, sepsis, and a presence of pain. When these camels presented for veterinary intervention, they were treated similarly to that described for llamas and alpacas [1]. Two of the cases were sedated, and their mandibular lesions incised and flushed. Both of those cases were managed with florfenicol, and one was also treated with flunixin meglumine. The last of the three cases was put under general anesthesia, and the lesion was treated surgically before being managed with flunixin meglumine and oxytetracycline postoperatively. All three cases had resolution of the mandibular infection.

In the literature, few references for mandibular region or tooth root-associated infections in camels exist. Some references may describe tooth root abscesses or similar infections, but the exact etiology of the case may be unclear. One case from 2004 identifies *Actinomyces viscous* in a dromedary camel presenting as bilateral mandibular exudation and necrosis [5]. The lesion was excised, and the camel was treated with 4 weeks of penicillin-streptomycin, with no progression noted at 24 weeks postoperatively [5]. A study of 61 cases of wounds to the head and neck of dromedary camels identified 8.2% (*n* = 5) with submandibular abscesses but was not specific to the agent or bony structure involvement [6]. In a study of 450 camels in Egypt (2001), 20 were diagnosed with cutaneous abscesses in the mandible or hind legs [7]. Culture of six of these from the mandible revealed 4 isolates of *Corynebacterium* species and one isolate each of *Staphylococcus* and *Streptococcus* [7]. These findings are similar to our first case where *Corynebacterium* and *Actinomyces*-like isolates were cultured. One of the isolates was *Corynebacterium pyogenes* (currently *Trueperella pyogenes* or also known as *Arcanobacterium pyogenes*), which was isolated in our second case. Although culture and susceptibility testing is vital to case management, due to the improvement of clinical data, the antimicrobial use in our reported cases seems reasonable, as *Corynebacterium* species are typically susceptible to beta-lactams, florfenicol, and sulfonamide antibiotics [8], and *Actinomyces* is commonly susceptible to florfenicol [9]. A recent case report identified mandibular actinomycosis in a llama that was refractory to antimicrobial treatment [10].

The basis of antimicrobial treatment for all three cases involved florfenicol. Florfenicol is a bacteriostatic protein synthesis inhibitor with broad-spectrum activity and a high volume of distribution, indicating potential for tissue penetration. More recent recommendations for South American camelids are for 3 treatments of 20 mg/kg administered every other day [1]. Clinicians should monitor cases where florfenicol is administered for extended periods of time, as dose-dependent bone marrow suppression has been described, although rarely, in hoof stock administered high doses of florfenicol [11]. Other recommended drugs that have also been described for dental infections in South American camelids are ceftiofur hydrochloride (2 mg/kg, every 24 hours for 14 days) and isoniazid (10 mg/kg, per os, every 24 hours for 30 days) [1]. Other drug candidates that could be explored for dental infections in camels could include tulathromycin. With a large volume of distribution and broad-spectrum activity [12], tulathromycin could be useful as it would also need to be administered infrequently, although more research is needed in the pharmacokinetics and pharmacodynamics of this drug in camels. One animal showed no improvement with oral administration of trimethoprim-sulfamethoxazole, while this may be an easier route of administration, the oral bioavailability of this drug is quite low in alpacas (7.7-10.5%) [13] and may not be useful if camels have similar bioavailability. If susceptible, sulfadimethoxine may be useful, as a study in llamas found an oral bioavailability of 52.6% [14]. Sodium iodide was used as an adjunctive therapy in this case series. Recent studies have suggested that sodium iodide supplementation may enhance the activity of the lactoperoxidase/hydrogen peroxide/iodide system and lead to pathogen inactivation in ruminant species [15]. It is uncertain if this enhancement occurs in camels, and more research is necessary for this determination.

Two of the camels in this case series received some form of therapy prior to presentation at the UTKCVM. While these therapies were initiated by veterinarians, they were performed with minimal diagnostic results prior to implementation. While the exact reasons for this are unknown, this could be due to an unfamiliarity with the species (as the North American population is estimated at approximately 5,000 animals [3]), or an untactable nature, as camels can be aggressive to manipulation in field settings.

Two of the patients in this case series were partially intractable during their hospitalization, which limited frequent administration of oral medications. Most of the patients included in this case series presented before meloxicam was used in a widespread fashion in livestock. Meloxicam is a nonsteroidal anti-inflammatory commonly used in cattle, sheep, goats, and South American camelids for pain management [16]. Intravenous pharmacokinetics of meloxicam in camels indicate extended clearance and a long elimination half-life [17, 18]. In llamas, these characteristics allow for clinical administration of oral meloxicam once every 3 days at 1 mg/kg dosing [19]. While the pharmacokinetics of orally administered meloxicam are not currently known for camels, oral administration of meloxicam tablets could provide for analgesia and anti-inflammatory therapy for these cases. This drug would only have to be administered once every several days, which could be convenient for camels which are not easily tractable.

Several etiologies have been proposed for dental disease progressing to infection in camelid species. Tooth root abscesses have been described in the New World Camelid literature [1]. Recently, a review of dental disease in alpacas has identified diastemata as a common finding that was associated with pulp exposure [20]. Diastemata is the finding of an abnormal space between the adjacent teeth. This irregular space can lead to materials accumulating and abnormal wear. These investigators also found in alpacas that mandibular swelling and periodontal disease were closely associated [21]. Due to the similarities in dental anatomy between Old World Camelids such as camels and New World Camelids such as alpacas, a comparative approach of routine dental examinations for abnormalities such as diastemata or mandibular swelling may be useful for identifying the early stages of dental disease in these species.

Radiographs are commonly used as the initial diagnostic test in large animal patients presented with head disorders including dental disease and were performed in all patients included in this case series. Radiographic equipment is readily accessible, the radiographic examination does not require general anesthesia, and the modality is adequate to demonstrate advanced abnormalities of the tooth roots and osseous structures especially if involving the lower dental arcade and ventral margin of the mandible. Early or subtle abnormalities and lesions affecting the upper dental arcade may be more challenging to diagnose radiographically. Clinicians should be prepared for sedation and/or anesthesia and consider advanced diagnostic imaging methods for complicated cases.

Based on the documented evidence that computed tomography (CT) scans can provide useful information when it comes to treatment of lesions progressing from tooth root abscessation to mandibular infections, this case series should guide future researchers in the direction of using advanced imaging techniques [1]. CT has proven superior to radiography in the evaluation of dental disease in horses and is now routinely used in this species [22]. Multiple research and clinical studies have investigated CT in the evaluation of normal and abnormal dentition and other maxillary abnormalities in New World Camelids [20, 21, 23–25]. While comparative studies in Old World Camelids are lacking to date, CT is expected to provide similar superior information to radiographs when evaluating these patients for suspected dental disease and may be considered in future clinical cases as well as research settings. Chronic dental infections may lead to a persistent bacteremia that may predispose animals to cardiac and joint infections. While this is known in other veterinary species, additional investigation will be necessary for this issue in camels.

In New World camels, tooth root infections can be managed surgically or medically [1]. For these three cases, one requires removal of the tooth, and the others did not. While medical management alone may not be enough for resolution of a similar infection in an Old World Camelid, it may be a reasonable initial therapy when the risks for anesthesia are considered. It is worth noting that lancing and flushing of the affected regions were utilized in all cases of this series. Additionally, due to the nature of the camels and limitations of handling, these cases do not have exhaustive descriptions of the oral examination. This would be useful information for future studies, as there may be sequelae or additional factors that were not appreciated.

This study's limitations include the small sample size and its retrospective nature. Additional limitations include the use of medical records that had been recovered from physical copy to digital (including radiographic reports and findings). While the study utilized records from three different camels seen at different times by different treatment teams, it is unlikely that the process for diagnostic workup and therapeutic treatment of mandibular infections in camels has changed significantly during the study period. This study also utilized camels in their non-native habitat (North America), and while the population of camels in North America appears to be increasing [3, 26], findings from this population may not be representative of camels in other regions.

In conclusion, it can be argued that successful treatment of mandibular infections in Old World Camelids can be achieved using methods previously described for New World Camelids (llamas and alpacas) [1]. The outcome of treatment in these three cases did not differ between medical management and surgical therapy. However, due to increasing North American population and dearth of data available, more research should be done on veterinary care with this species, as well as pharmacokinetics and pharmacodynamics of the drugs used to treat mandibular infections within the Old World Camelid group. It is also necessary to conduct additional large-scale and modern studies to assess and identify the microbial microbiota in these animals in the normal state of their physiological development, as well as in various oral disorders and diseases of the teeth and oral cavity. Until such further information is available, a comparative approach to mandibular infections of New World Camelids may be useful in the management of similar conditions in Old World Camelids, as supported by these three cases.

## Data Availability

All data is contained within the manuscript for this retrospective case series.
